# Optimizing Catalytic Depolymerization of Lignin in Ethanol with a Day-Clustered Box–Behnken Design

**DOI:** 10.1021/acs.iecr.2c03618

**Published:** 2023-04-28

**Authors:** Panos D. Kouris, Alberto Brini, Eline Schepers, Michael D. Boot, Edwin R. Van Den Heuvel, Emiel J.M. Hensen

**Affiliations:** † Laboratory of Inorganic Materials and Catalysis, Department of Chemical Engineering and Chemistry, Eindhoven University of Technology, Eindhoven 5600 MB, The Netherlands; ‡ Department of Mathematics and Computer Science, Eindhoven University of Technology, Eindhoven 5600 MB, Netherlands

## Abstract

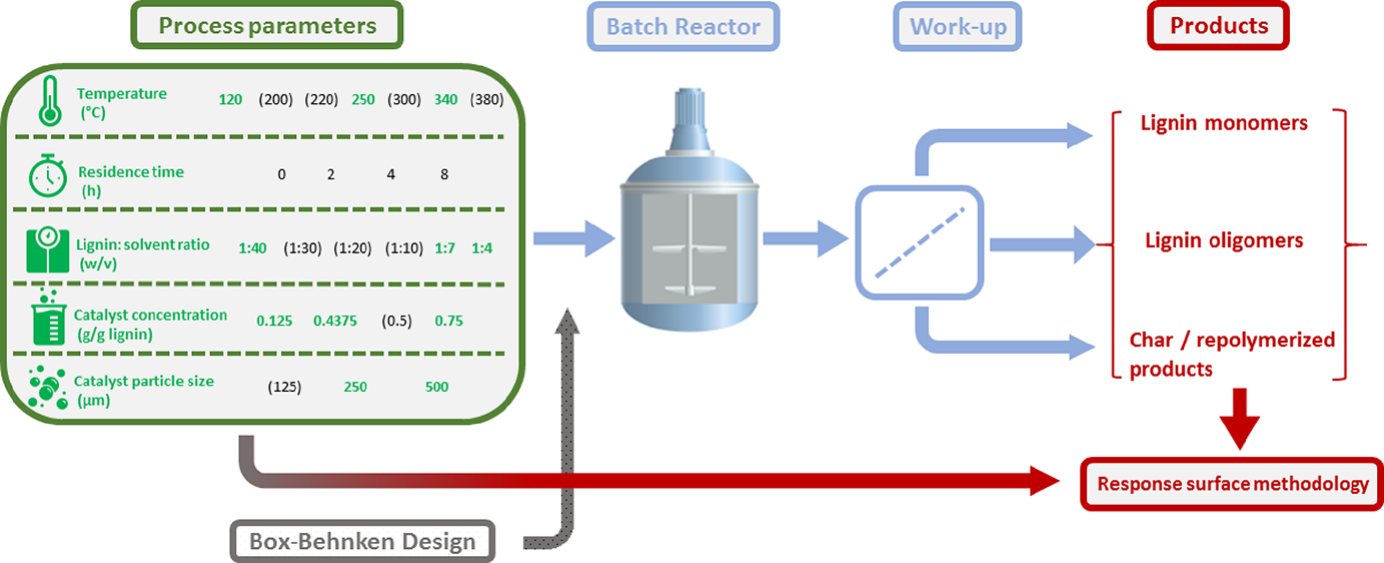

Lignin is a potential resource for biobased aromatics with applications in the field of fuel additives, resins, and bioplastics. Via a catalytic depolymerization process using supercritical ethanol and a mixed metal oxide catalyst (CuMgAlO_*x*_), lignin can be converted into a lignin oil, containing phenolic monomers that are intermediates to the mentioned applications. Herein, we evaluated the viability of this lignin conversion technology through a stage-gate scale-up methodology. Optimization was done with a day-clustered Box–Behnken design to accommodate the large number of experimental runs in which five input factors (temperature, lignin-to-ethanol ratio, catalyst particle size, catalyst concentration, and reaction time) and three output product streams (monomer yield, yield of THF-soluble fragments, and yield of THF-insoluble fragments and char) were considered. Qualitative relationships between the studied process parameters and the product streams were determined based on mass balances and product analyses. Linear mixed models with random intercept were employed to study quantitative relationships between the input factors and the outcomes through maximum likelihood estimation. The response surface methodology study reveals that the selected input factors, together with higher order interactions, are highly significant for the determination of the three response surfaces. The good agreement between the predicted and experimental yield of the three output streams is a validation of the response surface methodology analysis discussed in this contribution.

## 1 Introduction

Climate change due to emissions of CO_2_ from fossil resources together with the depletion of fossil reserves stimulates research into the conversion of renewable resources into sustainable fuels and chemicals. Lignin makes up to 15–30 weight (wt) % of lignocellulosic biomass and is as such the largest renewable source of aromatic building blocks for the production of bulk or functionalized aromatic compounds and fuels replacing petroleum feedstock.^1^ Lignin is a natural amorphous three-dimensional polymer consisting of methoxylated phenylpropane structures, cross-linked predominantly by C–O–C (β-Ο-4′, α-Ο-4′, 4-Ο-5′) and C–C (β–1′, β-β′, 5–5′) bonds.^2^ Over the past two decades, a wide variety of chemical treatment methods that aimed at breaking down lignin into smaller fragments has been explored.^1^ The main conversion routes include gasification, pyrolysis, and acid- or base-catalyzed oxidative or reductive depolymerization.^2^ Among these, reductive catalytic depolymerization is a promising method for obtaining fuel additives and aromatic chemicals because radical coupling reactions of intermediate fragments can be partially avoided by hydrogenation of reactive double bounds.^1,3^

In previous work by Huang *et al*., it was demonstrated that monomeric aromatics can be obtained in high yield from technical lignin using a mixed Cu–Mg–Al oxide catalyst in supercritical ethanol with little char formation.^4−7^ This approach was inspired by the methanol-mediated conversion of lignin using a similar catalyst as proposed by the Ford group.^8^ Ethanol not only acts as a hydrogen donor solvent but also as a capping agent to stabilize highly reactive phenolic intermediates by O-alkylation of hydroxyl groups and C-alkylation of aromatic rings. The monomeric products are mainly composed of alkylated aromatics and include phenols. The oxygen-free aromatics can be used as chemical building blocks and octane boosters when blended with gasoline,^9^ whereas oxygenated aromatics may serve as valuable compounds for the chemical and polymer industry.^10^ Additionally, they can also be used as soot suppressants in diesel fuel.^11^ This approach, designed to obtain mono-aromatics from a wide range of technical lignins, was however only demonstrated in a limited operating window of lignin and catalyst loadings. To be able to assess the commercialization potential of this technology, we need to expand the operational window and explore the potential for scale-up.

As a first step, Kouris *et al.* scaled up the process from a 100 to 4000 mL batch reactor, thereby identifying a trade-off between conversion and selectivity to monomers (i.e., lignin oil value drivers) and practical issues concerning high solvent dilution (i.e., lignin oil cost driver).^12^ Additionally, important capital expenditure (CAPEX) and operational expenditure (OPEX) indicators for the commercialization of this technology were evaluated. Several important aspects such as the influence of reaction temperature and lignin loading on the monomer yield, catalyst deactivation, and ethanol losses were also discussed. Catalyst fouling by deposition of heavy lignin fragments on the catalyst surface was the main reason for the low yields of mono-aromatics at high lignin-to-solvent feed ratios. However, during these investigations, we followed a traditional and straightforward experimental strategy to determine the relationships between factors (e.g., temperature and lignin:solvent ratio) and the response of the process, namely, the one-factor-at-a-time (OFAT) approach. The OFAT method includes selecting baseline levels for each factor followed by successively varying each factor across a range of interest, while the other factors are held constant at their baseline levels. A significant disadvantage of this approach is that it does not consider any possible interactions between the selected factors.

**Figure 1:**
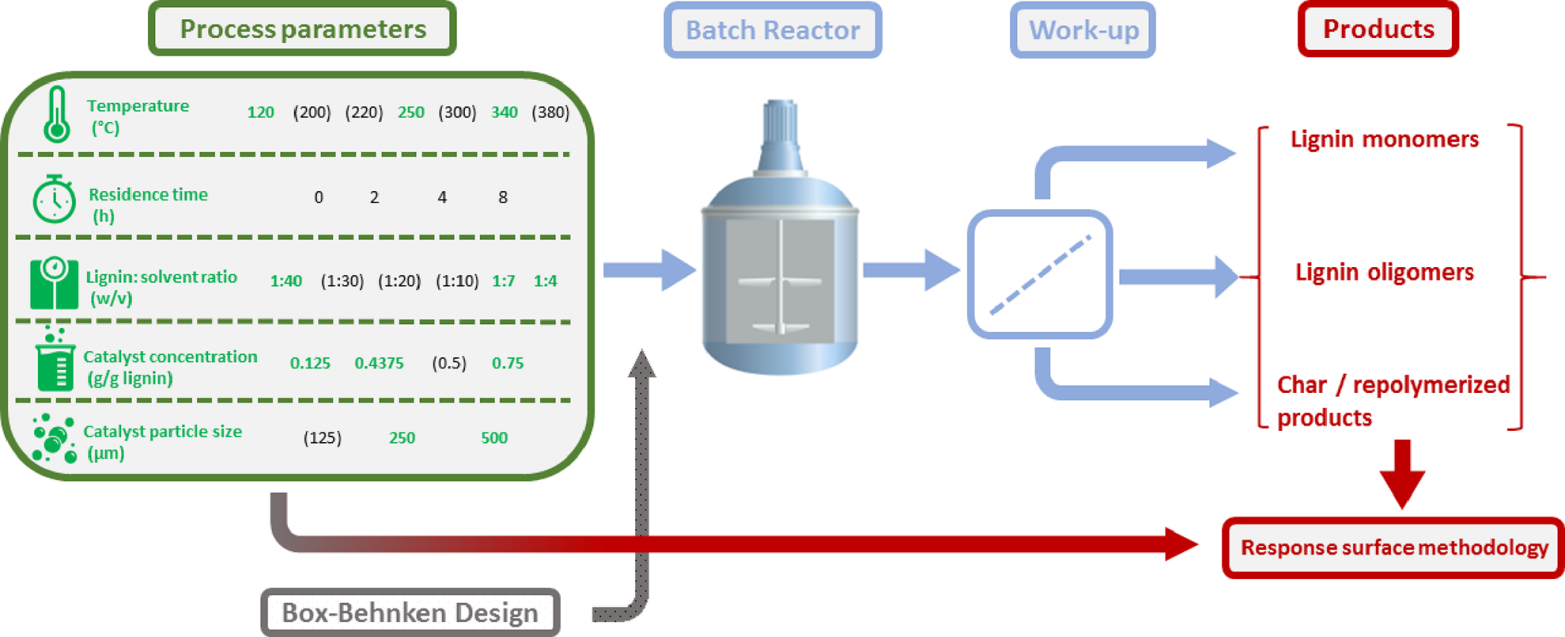
Schematic overview of the investigated process parameters utilized by the Box–Behnken method for an optimal experimental design. The response surface methodology was employed to evaluate the effects of process factors, identify the relevant interactions among different process parameters, and eventually search for optimum operating conditions. The process parameters in black were evaluated in previous studies by Huang *et al.*(4−7) and Kouris *et al.*,^12^ while those in green represent the selected process window of the present study.

In the present contribution, we shed more light on the trade-off between yield optimization and cost minimization and, thus, the techno-economic viability of the proposed lignin upgrading technology, by exploring a wider range of relevant process parameters impacting the product yield and distribution (Figure 1). We consider catalyst concentration and particle size as well as reaction time as the main process parameters. Catalyst concentration and particle size can provide insights into possible mass transfer limitations during lignin depolymerization. Reaction time in combination with operating temperature is a crucial factor that can influence specific chemical routes, such as lignin solubility, depolymerization, deoxygenation, or undesired side condensation reactions. This new set of process parameters, together with reaction temperature and lignin-to-solvent ratio, along with their interactions, are important performance criteria for industrial operation.

We apply for the first time the Box–Behnken design (BBD) and the response surface methodology (RSM) to evaluate the process performance of our technology and propose optimum process conditions for commercialization. The RSM, which represents a collection of statistical tools for designing experiments, can evaluate the impact of parameters, predict the interaction among them, and eventually search for optimum operating conditions. There is limited work that combines the RSM with the structured design of experiments for the exploration of lignin depolymerization processes. Gasser *et al.*(13) reported a novel sequential lignin treatment method consisting of a biocatalytic oxidation step followed by a formic acid-induced lignin depolymerization step and optimized RSM. The RSM was used to study the effect of five parameters on lignin reaction products, i.e., enzymatic activity, substrate concentration, enzymatic treatment time, initial pH, and reaction time, under a Q-optimal design. Being a minimum level (i.e., near-saturated) design, such design cannot evaluate the sufficiency of a quadratic model, and therefore, it must be augmented to test for a lack of fit. Similarly, Jung *et al.*(14) studied the bio-oil production of lignin pyrolysis in a fixed-bed system, which was investigated through an RSM to optimize a restricted set of operating variables such as temperature, heating rate, and loading mass, under a BBD. According to the mathematical model of the RSM, the temperature was identified as the most significant variable of the process and the maximum bio-oil yield could be predicted at the optimum reaction temperature.

Herein, we focus on providing more insight into the above-selected process parameters of the lignin ethanolysis and their impact on the desired reaction products. Specifically, we use Design of Experiments (DoE) to carry out measurements by applying randomization, replication, and blocking of the process parameters under investigation. The experimental runs were determined using a BBD,^15^ a second-order multivariate technique based on three-level partial factorial designs that permits the estimation of the parameters of a quadratic model and a consequent investigation of a lack of fit in the model. The substantial amount of process parameters resulted in 108 experimental runs that were properly combined in 81 experimental blocks to reduce the number of days required to perform the measurements. We employ the RSM as an analytical tool for our investigation to determine a quantitative relationship between the process conditions and the yields of the various product streams (i.e., monomers, oligomers, and repolymerized products), with the application of linear mixed models (LMMs).^15^ The use of LMMs for the analysis of unbalanced designs has been adopted in practice to accommodate the imbalance derived from practical issues or limitations given by technical instrumentation.^16^ We will refer to this approach as LMM-RSM throughout this chapter.

## 2 Methods

### 2.1 Experimental Methods

#### 2.1.1 Chemicals and Materials

Protobind 1000 alkali lignin was purchased from GreenValue, which is produced by means of soda pulping of wheat straw. This particular lignin is void of sulfur and contains only minor amounts of impurities (e.g., <4 wt % carbohydrates and <2 wt % ash). All commercial chemicals were analytical reagents and were used without further purification.

#### 2.1.2 Catalyst Preparation

A 20 wt % Cu-containing MgAl mixed oxide (CuMgAlO_*x*_) catalyst was prepared by a co-precipitation method with a fixed M_2+_/M_3+_ atomic ratio of 4. CuMgAlO_*x*_ (100 g) was prepared in the following way: 50.87 g of Na_2_CO_3_ was solved in 300 mL of deionized water, filled into a 2 L beaker, and then warmed to 60 °C. NaOH solution (500 mL, 50 wt %) was subsequently poured into a 500 mL dropping funnel. In parallel, 70.71 g of Cu(NO_3_)_2_·2.5H_2_O, 332.30 g of Mg(NO_3_)_2_·6H_2_O, and 150.05 g of Al(NO_3_)_3_·9H_2_O were dissolved in 500 mL of deionized water and poured into a 500 mL dropping funnel. The aforementioned two solutions were then slowly added to the 300 mL Na_2_CO_3_ solution while stirring and keeping the pH of the slurry at 10. Next, the precipitate was filtered and washed until the filtrate reached a pH of 7. The solid was then dried overnight at 105 °C and sieved to different particle sizes for the purpose of this work. Finally, the hydrotalcite structure of the obtained powder was calcined at a heating rate of 2 °C/min from 40 to 460 °C and kept at this temperature for 6 h in static air. The resulting catalyst was denoted by Cu_20_MgAl(4).

#### 2.1.3 Catalyst Characterization

The metal content of the CuMgAlO_*x*_ catalyst was determined by inductively coupled plasma optical emission spectroscopy (ICP-OES) with end on plasma (axial plasma) viewing on a SPECTROBLUE EOP spectrometer with 165–177 nm wavelength range. All samples were dissolved in a mixture of H_2_O and H_2_SO_4_ (1:1 v/v) and prepared in duplo.

#### 2.1.4 Lignin Conversion

A 100 mL Amar autoclave was charged with a suspension of catalyst and lignin (amounts according to the BBD, see Section 2.3.) in 40 mL of ethanol. *n*-Dodecane (10 μL) was added as the internal standard. The reactor was sealed and purged with nitrogen several times to remove oxygen. The pressure was set to 20 bar, and the reactor was tested for leaks. The reaction mixture was heated to the desired temperature (according to the BBD, cf. Section 2.3) under continuous stirring at 500 rpm. The reaction time was started when the desired temperature was reached, and then, the reactor was left for the desired number of hours. After the reaction, the heating oven was removed, and an aliquot of 1 mL was taken from the reaction mixture for GC–MS analysis. The reactor was allowed to cool down to room temperature in an ice bath, and a gas sample was taken at room temperature for analysis.

#### 2.1.5 Workup Procedure

**Figure 2:**
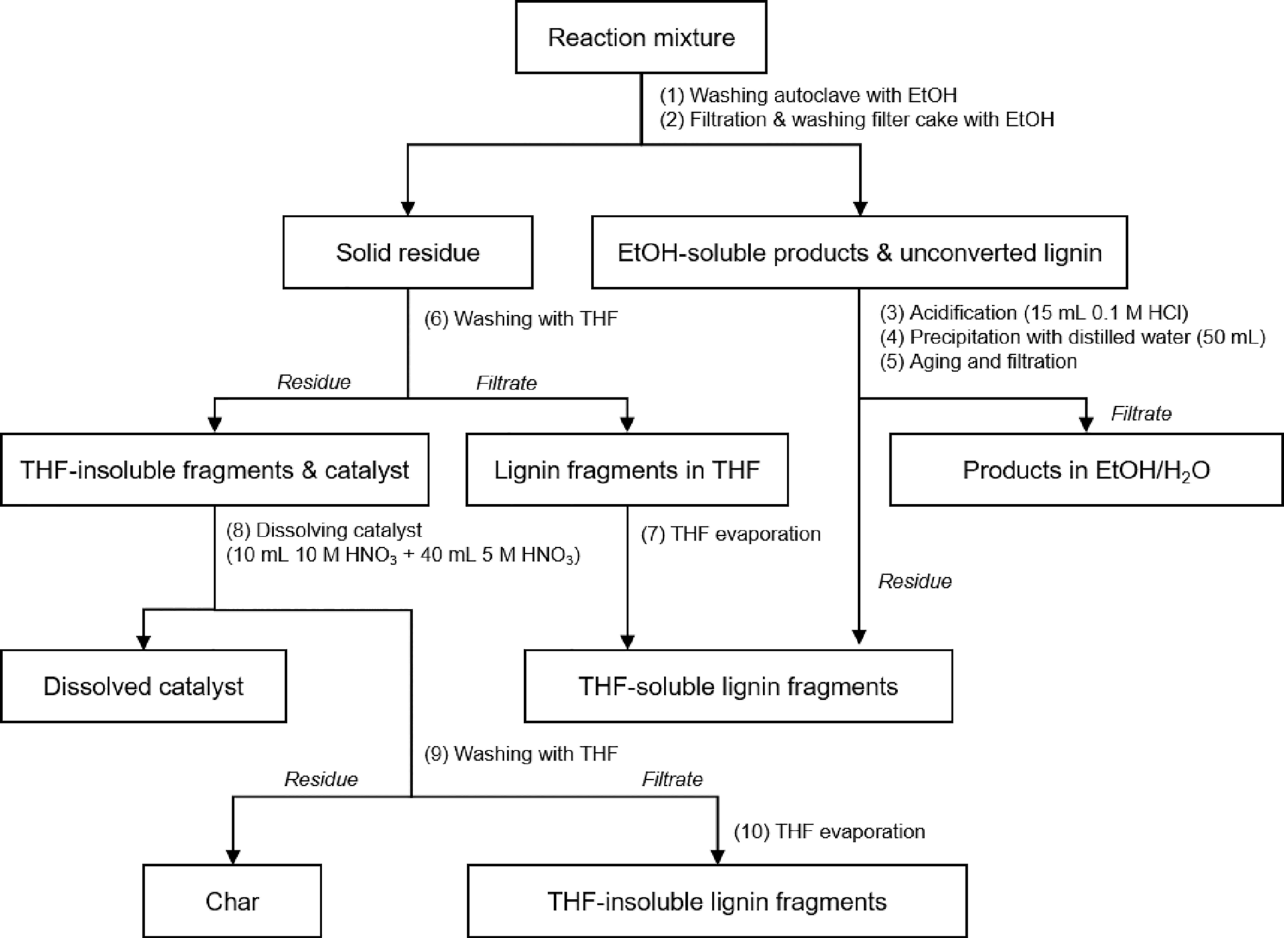
Workup procedure of the reaction product mixture.

A general workup procedure was used for further processing of the reaction mixture, based on the procedure developed in previous work from Huang and co-workers (Figure 2). The reaction mixture was collected, and the autoclave was washed with ethanol (1). Both the reaction mixture and the obtained solution after washing were combined. The combined mixture was filtered over a filter crucible (porosity 4), and the filter cake was washed with ethanol several times (2). The filtrate was acidified by adding 15 mL of a 0.1 M HCl solution (final pH = 1) (3), and 50 mL of distilled water was added to precipitate unconverted lignin and lignin fragments (4). The mixture was aged for approximately 30 min, after which it was filtered over a filter crucible (porosity 4) (5). The filter cake from (2) was washed with excess THF (6), after which THF was removed from the filtrate by rotary evaporation at 60 °C (7). The residues from (7) and (5) were combined and denoted as THF-soluble lignin fragments. The residue from (6) was denoted as a mixture of catalyst and THF-insoluble lignin fragments.

#### 2.1.6 Catalyst Dissolution

To identify the nature of the THF-insoluble lignin fragments, the catalyst was dissolved by adding 10 mL of 10 M HNO_3_ to 200 mg of solid residue from step (6) to dissolve copper followed by addition of 40 mL of 5 M HNO_3_ solution (8). The mixture was filtered over a filter crucible (porosity 4), and the filter cake was washed with excess THF (9). The remaining residue from (9) was regarded as char, and THF was evaporated from the obtained filtrate by rotary evaporation (10). The residue was denoted as THF-insoluble fragments.

### 2.2 Product Analysis

#### 2.2.1 Gas Chromatography–Mass Spectrometry (GC–MS)

The liquid product mixture after the reaction was analyzed on a Shimadzu QP2010 SE GC–MS apparatus, equipped with an RTX-1701 column (60 m × 0.25 mm ID × 0.25 μm), and a flame ionization detector (FID) together with a mass spectrometer detector. GCMSsolution and GCsolution software were used for the analysis. Products in the liquid phase were identified based on a search of the MS spectra in the NIST11 and NIST11s MS libraries. The quantitative analysis of the liquid phase products was based on the GC-FID measurements. The FID weight response factors of all product compounds in the liquid mixture were determined using the Effective Carbon Number (ECN) concept relative to *n*-dodecane as the internal standard. The yields of monomers, oligomers, and char were determined according to eqs 1–3:
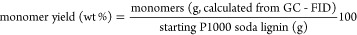
1
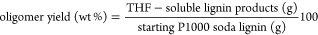
2
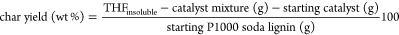
3Char yield is defined as a combination of THF-insoluble lignin fragments and actual char, as was collected during the workup procedure after step (6) (Figure 2).

#### 2.2.2 Gel Permeation Chromatography

GPC analyses of product fractions were performed on a Shimadzu Prominence-I LC-2030C 3D apparatus, equipped with two columns (Mixed-C and Mixed-D, Polymer Laboratories) in series and a UV–Vis detector at 254 nm. The columns were calibrated with polystyrene standards, and analyses were performed at 25 °C using THF as an eluent. Samples were prepared at a concentration of 2 mg/mL in non-stabilized THF and then filtered using a 0.45 μm filter membrane.

### 2.3 Experimental Design and Statistical Modeling

A BBD was selected such to fit a second-order response surface model. The class of design is based on the construction of balanced incomplete block designs, which pair together two out of the available factors in a 2^2^ factorial, while the other factors remain fixed at the center. The BBD also requires three evenly spaced levels of each factor. This structure ensures that there is sufficient information available for testing the lack of fit. For instance, when four factors are considered, the use of six center points would allow five degrees of freedom for the pure error and 11 degrees of freedom for the lack of fit.

**Figure 3:**
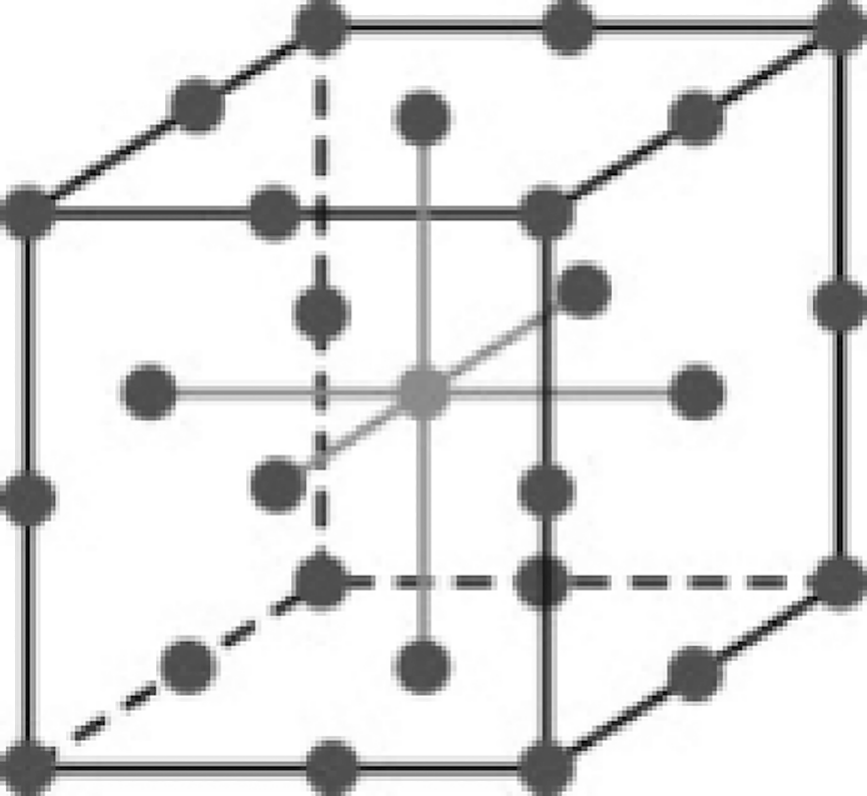
Visualization of a three-factor combination Box–Behnken design.

A BBD is a spherical design, which means that all edge points are at a constant distance from the design center. This implies that some of the extreme settings of the process parameters might not be covered. The analyst should not view this lack of coverage as a reason to discourage the use of a BBD but rather to highlight the fact that this design should be adopted in situations when there is no interest in predicting responses at the extremes. BBDs are also (nearly) rotatable, which ensures that a stable quality of the predictions of future responses is achieved throughout the region of interest.^15^Figure 3 shows an example of the location of each factor-level in a three-factor design.

Preliminary experiments, in our previous work, aided to establish the range over which these factors were explored. The lignin-to-ethanol ratio (w/v) was explored across a wide range (1:40 w/v to 1:10 w/v) to examine the impact on the yield of lignin monomers and ethanol losses.^12^ The range for lignin loading was established based on performance criteria for industrial operation. The reaction temperature range was initially balanced between 120 and 380 °C. The upper limit was established based on the previous findings by Huang *et al*.^4,7^ At 380 °C, depolymerization reactions are enhanced, mostly because thermal cracking starts and the more recalcitrant bonds in typical lignins can be cleaved. Also, hydrogen evolution via ethanol reforming, which promotes hydrogenolysis reactions, are dominant at high temperatures (340–380 °C). However, the reactor in use later limited the exploration of such an input factor to 340 °C. The updated process parameter violated the orthogonality hypothesis of the BBD and introduced potential correlations between the process parameters. A small simulation study was performed to assess the impact of the change in the design of the temperature setting. A synthetic outcome was simulated with a standard normal distribution and fitted according to eq 4 using the same input settings of our case study, i.e., the center point of the variable temperature was shifted to 0.182. Since the correlation matrix of the estimated model parameters showed non-zero values in the off-diagonal elements of moderate size (≤0.25 in absolute value), in the interaction effects of temperature with lignin-to-ethanol ratio, catalyst concentration, and particle size, no further steps were taken in this concern.

The summary of the factor-level combinations are shown in Table 1. Three response variables were selected to evaluate the influence of each of the five factors on the reaction process. Of the three variables, two were desired to be maximized: lignin monomers and THF-soluble fragments, whereas the THF-insoluble fragments were to be minimized. These three output factors were monitored at four different evolution stages of the reaction, i.e. 0, 2, 4, and 8 h. The total number of experiments for this study was therefore 108 (27 × 4), where for each time point, we have three experimental runs at the center points.

**Table 1:** Factor-Level Combinations for BBD of Experiments

	levels		
factor	–1	0	1
temperature (°C)	120	250^a^	340
lignin:solvent (L:S) ratio (g/mL ethanol)	1:40	1:7	1:4
catalyst concentration (g/g lignin)	1:8	7:16	3:4
catalyst particle size (μm)	125	250	500

a Adapted center due to the modified extreme value.

In experimental design, randomization of order presentation is generally used to avoid any confounding factors that might result from the specific presentation order. In this analysis, a day-clustered randomization was proposed to minimize the number of days to complete the experiments and maximize the number of runs that could be performed on a working day. For instance, an 8 h experimental run needed about 10 h for its preparation and execution. Therefore, a working day could consist of either a random combination of a 0 h and 2 h experiment or a single 4 or 8 h experiment. These working day blocks were randomly chosen, and their order was then shuffled. This procedure reduced the number of days needed for experimentation from 108 to 81 but could have made the results within the block (i.e., day) more alike than results across the blocks.

The collected results were entered into SAS statistical software, version 9.4, for statistical analyses. Each response variable was fitted according to two different LMMs: the first one models the reaction time as a continuous variable with a second-order polynomial profile:

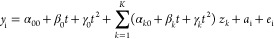
4
where *y*_i_ is the measured output (i = 1:3), α_00_ is the intercept, β_0_ and γ_0_ are the linear and the quadratic effect sizes of the reaction time, α_*k*0_ is the effect size of the corresponding covariate *z_k_* (including linear *x_j_*, interaction *x*_*j*_1__*x*_*j*_2__, and quadratic *x*_*j*_^2^ terms) coded as in Table S2. The two random effects *a*_i_ and *e*_i_ are standard normal distributions with variances τ_i_^2^ and σ_i_^2^, accounting for the day-to-day or block-to-block variation (i.e., the systematic error introduced in the day-clustered randomization) and the residual (i.e., unexplained) variability of the response-surface model, respectively. Note that eq 4 includes a linear profile for the reaction process when γ_0_ = 0. We will refer to the model in eq 4 as the "Continuous Time" model.

The second model considers a more general time-profile pattern for the reaction time, where each time assessment has its own specific level (i.e., time is treated as a categorical variable), as follows:
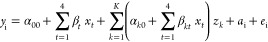
5where β_*t*_ is the effect size of the reaction at time *t* and β_*kt*_ is the coefficient of the interaction between the reaction at time *t* and the covariate *z_k_* (including linear *x_j_*, interaction *x*_*j*_1__*x*_*j*_2__, and quadratic *x*_*j*_^2^ terms). For the upcoming discussions, we will denote the model in eq 5 as the "General Time" model. Both models were estimated with maximum likelihood estimation using the procedure MIXED in SAS 9.4. The best time profile (continuous or categorical) was selected from the optimal mean structure by a goodness of fit analysis; i.e., AICc (corrected Akaike information criterion) and BIC (Bayesian information criterion). The model with the time profile that has the lower AICc and/or BIC has been selected. Then, a backward elimination approach was performed on the selected model to determine the optimal model for each output variable: the non-significant input parameters were eliminated from eq 4 or eq 5 always preserving the hierarchical structure. The order of elimination was determined using the Type3 *F* statistic, with a significance level set at 0.1 (10%). The final model was fitted using a restricted maximum likelihood (REML) estimation to ensure a better estimation of the variance components. The goodness of fit of the final model was evaluated through a regression analysis of the predicted vs the observed response of each output stream. The R square index has been used as an evaluation index of the adequacy of each LMM. Under the standard assumptions of LMMs, it is expected that the residuals follow a normal distribution. A Shapiro–Wilk test has been used to check for the normality of the studentized residuals, i.e., standardized residuals with equal variance.^17^

## 3 Results and Discussion

### 3.1 Qualitative Analysis

#### 3.1.1 Influence of Lignin Loading and Reaction Temperature

Earlier, we demonstrated a technology at both lab and bench scales able to obtain predominantly mono-aromatics from technical lignin using a CuMgAl mixed oxide catalyst.^4−7,12^ In the present study, we explore and evaluate the catalytic performance for soda lignin conversion in ethanol at a lab scale in a 100 mL autoclave by varying the lignin:solvent feeding ratio and reaction temperature, reaction time, catalyst concentration, and catalyst particle size. This technology as well as most of the related endeavors meets a common and glaring problem, i.e., the small lignin-to-solvent ratio in a batch reactor, which severely limits the efficiency of lignin conversion. Usually, only 10–50 mg of lignin is depolymerized per unit volume (1 mL) of solvent.^1,8,18,19^ Unfortunately, the literature often focuses on the chemistry of the conversion to monomers and the optimum catalyst design required to achieve it. While interesting from a scientific perspective, such dilute feeds would require an unrealistically high capital expenditure (CAPEX), which scales quite well with the total mass flow through the plant.

**Figure 4:**
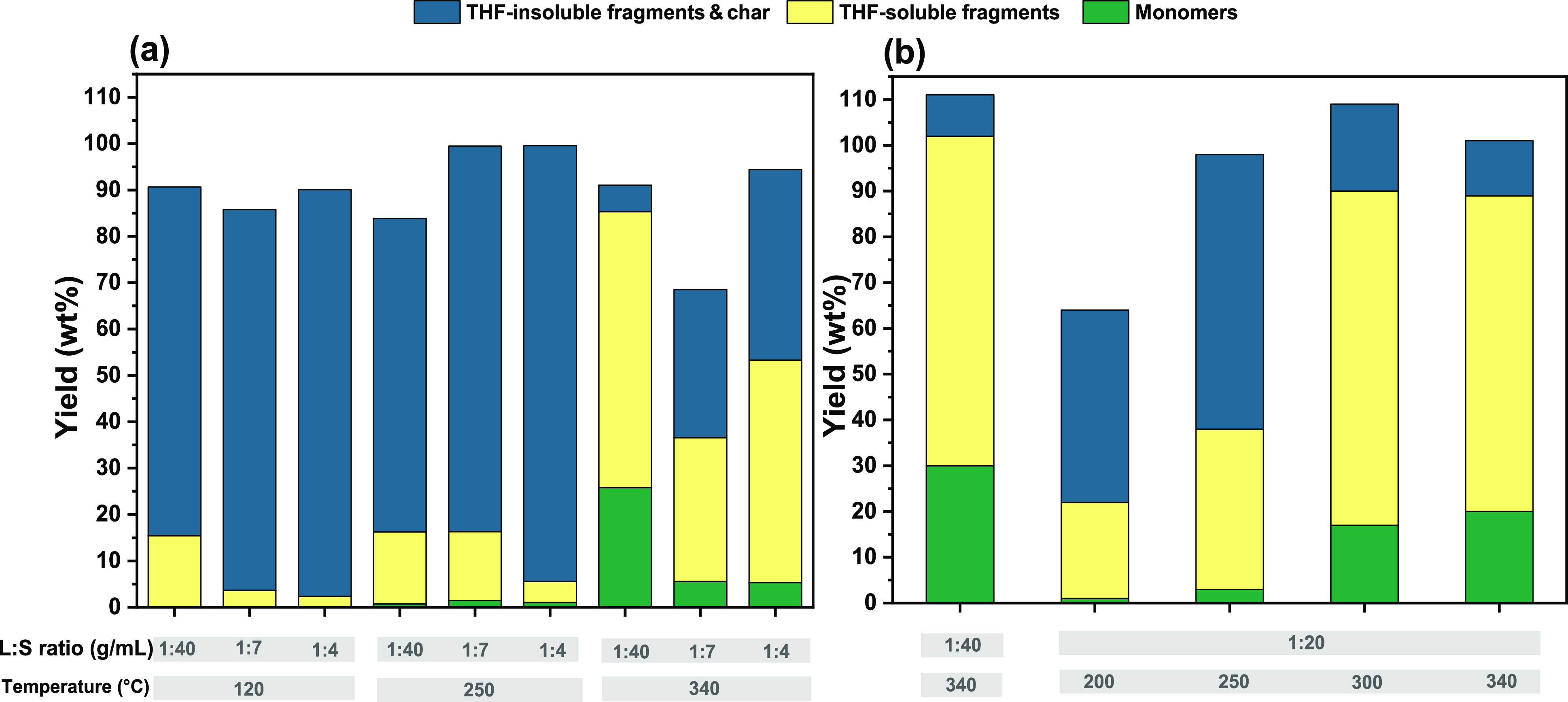
Yield of monomers, light residues, and heavy residues for different lignin:solvent (L:S) ratios (g/mL) across the reaction temperatures of catalytic depolymerization of Protobind soda lignin over the Cu_20_MgAl(4) catalyst for 4 h at a fixed catalyst concentration (0.4375 g cat./g lignin) and catalyst particle size (125 μm). (a) Current work and (b) work by Huang *et al*.^7^

Figure 4a illustrates some of the catalytic results for soda lignin conversion in ethanol on the effect of various lignin:solvent (w/v) ratios and reaction temperatures for a fixed residence time (4 h), catalyst concentration (0.4375 g cat./g lignin), and catalyst particle size (125 μm). A post-reaction workup procedure was developed to distinguish not only the lignin monomers but also smaller (tetrahydrofuran (THF)-soluble) and larger (THF-insoluble) lignin fragments and char.^5^ The THF-soluble residue contains lignin fragments with a lower molecular weight than the original lignin. The THF-insoluble residue is strongly adsorbed on the solid catalyst. After digesting the solid catalyst in nitric acid, this solid fraction becomes THF-soluble and its molecular weight can be identified. Char is characterized by the fraction that is strongly adsorbed to the solid catalyst and cannot be washed away by THF after catalyst dissolution.

Our results demonstrate that at 120 °C, hardly any lignin monomers could be obtained and the lignin solubility in ethanol was poor for all three selected lignin loadings. A yield of 16 wt % soluble lignin fragments was obtained when the diluted version of lignin-to-ethanol ratio (1:40 w/v) was employed. When we increased the lignin loading to 1:7 and 1:4 w/v, only 3 and 2 wt % yield of THF-soluble fragments could be achieved, respectively. On the other hand, high yields of THF-insoluble fragments and char, adsorbed on the solid catalyst, were identified for all three lignin loadings. A yield of 75 wt % insoluble lignin fragments was acquired for the 1:40 w/v lignin:ethanol ratio, while at higher lignin loadings, these yields were in the range of 87–90 wt %. At a higher reaction temperature (250 °C), the amount of solubilized lignin fractions remained at a similar level for the lowest lignin loading (15 wt %), slightly decreased to 14 wt % for the 1:7 w/v lignin:ethanol ratio, and decreased even further for the highest lignin loading (4 wt %). We observed that a small amount of lignin monomers with values in the range of 0.7–1.4 wt % was produced. The large and ethanol-insoluble lignin compounds were again the dominant solid fraction ranging from 67 to 94 wt % across all lignin loadings. Thermal solvolysis of lignin is an essential step during catalytic depolymerization. First, solid lignin should be solubilized and fractionated by the aid of the solvent into low-molecular-weight lignin fractions, which accordingly can be adsorbed onto the catalytic active sites for further depolymerization to mono-aromatics. The yield and the quality of the solubilized fractions depend on the reaction temperature, residence time, and lignin loading. Earlier, we investigated the solvolysis of Protobind lignin (without a catalyst) toward the formation of oligomeric fractions by varying residence time and reaction temperature.^20,26^ The temperature was varied from 25 to 200 °C, while the residence time and lignin:ethanol ratio were 30 min and 1:5 w/v, respectively. It was found that the yields of solubilized lignin at 25 °C were 12.7 and 49.8 wt % at 120 °C and finally reached maximum conversion of 56 wt % at 200 °C. Prolonged reaction times did not influence the solubility yields but only the quality of the soluble fractions since repolymerization reactions occurred. Lignin oligomers can though repolymerize by depletion of ether bonds and formation of carbon–carbon bonds.^21−25^ These results do not agree with the results of the catalytic ethanolysis of lignin of the present work with low yields of THF-soluble lignin fractions obtained at 120 and 250 °C for all three lignin loadings. An explanation can be that prolonged reaction times at these temperatures lead to enhanced condensation reactions, which yield to non-soluble high molecular weight (*M*_w_) products.

**Figure 5:**
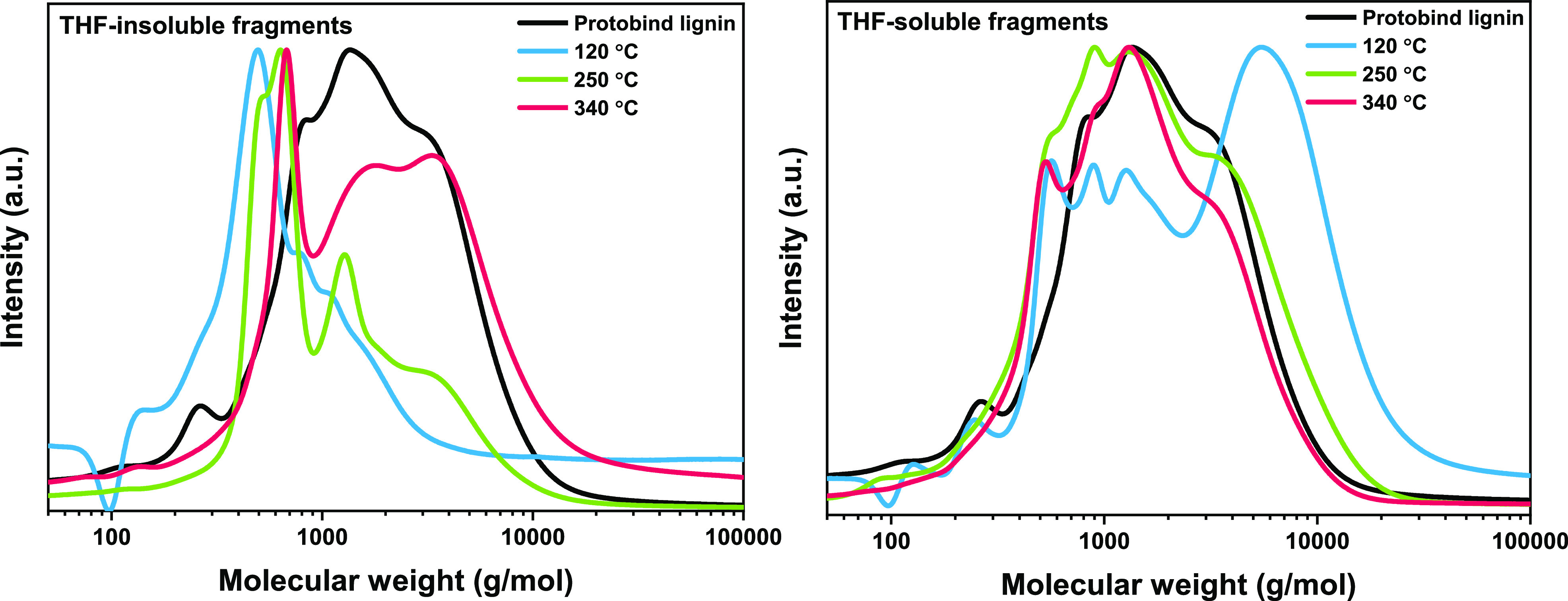
Molecular weight distributions of Protobind lignin (THF-insoluble fraction) (left) and THF-soluble fractions (right) obtained after reaction at different temperatures for a lignin loading of 1:7 w/v, catalyst concentration of 0.4375 g/g lignin, and catalyst particle size of 125 μm over the CuMgAl mixed oxide catalyst. (Depicted chromatograms have been normalized).

We characterized by GPC the THF-soluble and THF-insoluble lignin fractions for different reaction temperatures and for a constant lignin:ethanol ratio (1:7 w/v), residence time (4 h), catalyst concentration (0.4375 g/g lignin), and catalyst particle size (125 μm). The normalized gel permeation chromatograms are shown in Figure 5. For comparison, the parent lignin material was also analyzed using THF as a solvent. The *M*_w_ of this fraction is 1100 g/mol. Compared to the Protobind lignin, the increased signal in the lower *M*_w_ area (500 g/mol) of the THF-insoluble product stream after reaction at 120 °C points to the formation of depolymerized lignin fragments. Probably, thermolytic reactions take place at low temperatures, resulting in fragments of lower molecular weight that are adsorbed on the catalyst surface, as a result of the strong adsorbing nature of lignin. At these high lignin loadings, the catalyst surface is possibly poisoned with lignin fragments that are difficult to desorb from the surface at low temperatures due to lack of energy. At a higher temperature (250 °C), the same behavior was observed. After catalyst dissolution, adsorbed lignin fractions with lower *M*_w_ than the parent lignin could be determined (550–670 g/mol). Next to that, a shoulder at the high-*M*_w_ area develops in the gel permeation chromatogram of the THF-insoluble lignin fragments. This effect is more prominent for the highest reaction temperature (340 °C). It is clear that condensation products are formed that remain adsorbed on the catalyst with increasing temperature. This is in agreement with the results obtained in previous work by Huang *et al*. for lower lignin loadings.^4,6,7^ The *M*_w_ distributions of the THF-soluble product streams at 250 and 340 °C were comparable to the previous results of Huang *et al.* when a lower lignin loading (1:20 w/v) was used. Based on Figure 5, we observe that depolymerization occurs in that temperature range since the determined *M*_w_ of the soluble products at 340 °C was 628 g/mol. However, when looking in more detail at the molecular weight distributions of the THF-soluble products at 120 °C, it was found that this stream contains a significant amount of products with molecular weights in the range of 6000–7000 g/mol. These products could be a result of condensation reactions of reactive lignin fragments for long reaction times as repolymerization suppression reactions (i.e., alkylation, Guerbet, and esterification) do not have sufficient rates at low temperatures.

The yields of THF-soluble fragments were slightly increased at the reaction temperature of 250 °C, as shown in Figure 4a. Comparison at a temperature of 250 °C of the data for the various lignin:solvent ratios reveals that the solubilized oligomeric fractions for the lowest lignin loading was only 16 wt %, while for an intermediate loading (1:7 w/v), the THF-soluble fraction reached 15 wt %. At an extreme lignin:ethanol ratio (1:4 w/v), the yield of THF-soluble fractions declined to 5 wt %. In previous work by Huang *et al*.,^7^ the effect of temperature on the product yields was studied at low lignin loadings (Figure 4b), allowing to conclude that condensation reactions are dominant at low reaction temperatures (200–250 °C), leading to more THF-insoluble fragments. Furthermore, it was found that, at elevated reaction temperatures (300–340 °C), depolymerization reactions were enhanced, which resulted in a decreased yield of THF-insoluble products and an increased yield of THF-soluble fragments and monomers. The current work confirms these earlier findings but only for the lowest lignin loading employed herein. Higher lignin loadings are limiting the solvolytic efficacy of ethanol. We suspect that the insoluble fractions that were formed during the previous operating temperature (120 °C) accumulate in the reaction system, increasing significantly the THF-insoluble and char fragments on the catalyst surface.

**Figure 6:**
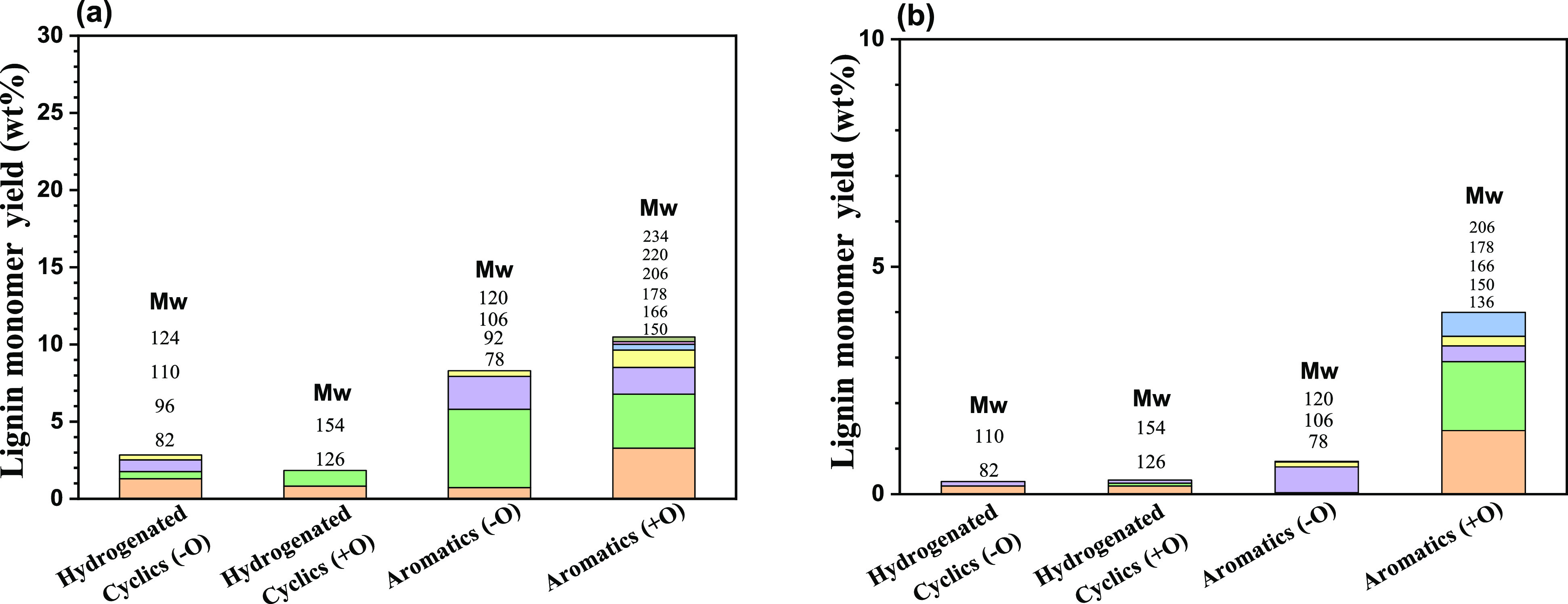
Lignin monomer distribution from Protobind lignin conversion at 340 °C using the Cu_20_MgAl(4) catalyst for 4 h at a fixed catalyst concentration (0.4375 g cat./g lignin) and particle size (125 μm) at (a) 1:40 w/v and (b) 1:4 w/v lignin:ethanol feeding ratios.

On the other hand, at 340 °C, a higher hydrogen pressure is achieved that facilitates the hydrogenolysis reactions, which effectively improves the lignin monomer yield. A monomer yield of 25 wt % was obtained for the lignin:ethanol ratio of 1:40 w/v. Figure 6a shows the typical lignin-derived product distribution of the monomer fraction of the products obtained after reaction at 340 °C for 4 h over the Cu_20_MgAl(4) catalyst. The primary products were aromatics with hydrogenated cyclic products as the main side products. Most of these products were alkylated with methyl and/or ethyl groups substituted on the rings. At that temperature, the lignin monomer yield was improved due to more efficient thermocatalytic and thermal cracking of the highest recalcitrant fraction of lignin; a fact that is also confirmed by the increased THF-soluble oligomeric fractions. A further increase in the lignin loading to 1:7 and 1:4 w/v resulted in poor monomer yields, manifesting mainly in the thermal cracking of some weak ether bonds and not in hydrogenolysis reactions on the catalytic sites since these are blocked by adsorbed species (as confirmed by GPC, Figure 5). According to the gas-phase analysis presented in Table S1, low amounts of H_2_ produced in the ranges of 0–50 and 160–200 g/mL were observed at low (120 °C) and moderate (250 °C) reaction temperatures, respectively. The low yields of monomers at these temperatures (Figure 4) are likely due to the low rate of ethanol reforming, explaining the low hydrogen concentrations and limited lignin hydrogenolysis reactions. On the other hand, at a temperature of 340 °C, higher hydrogen concentrations in the range 200–460 g/mL were observed at different reaction times. Figure 6b presents the distribution of the monomer fraction of the products obtained after reaction at 340 °C for the 1:4 w/v lignin:ethanol ratio over Cu_20_MgAl(4), which corresponds only to 5 wt %. The THF-insoluble lignin residue was the dominant solid fraction together with the THF-soluble lignin fragments. However, the large amount of heavy fragments adsorbed onto the catalyst surface cannot allow the oligomeric fractions to access the active sites and contribute to higher monomer yields.

### 3.2 Analysis of the Response Models

Several qualitative relationships between the input variables and the products have been described in the previous section. In this section, we provide a collection of all the results obtained from the LMM-RSM analyses applied to our set of 108 experimental runs to investigate all the interactions among all input parameters. Table 2 collects the goodness of fit analyses for the choice of the time profile to be adopted in LMM-RSMs for the three response variables (yield of monomers, yield of THF-soluble fragments, and yield of THF-insoluble and char fractions). The predicted values obtained from each LMM are compared with the actual values from the experimental studies to evaluate the consistency and the acceptability of the theoretical model fitted on the individual responses.

**Table 2:** Goodness of Fit for the Choice of the Time Profile in Equations 4 and 5^a^

	output					
	yield of monomers		yield of THF-soluble fragments		yield of THF-insoluble fragments and char	
info criteria	AICc	BIC	AICc	BIC	AICc	BIC
general time model	538.8	544.7	875.0	871.6	801.4	815.6
continuous time model	522.2	537.0	843.7	860.0	832.3	816.0

a For AICc and BIC, a lower measure points to a better fit.

The application of LMM-RSM yielded regression equations collected in Table S2, representing empirical relationships between the yields of monomers, small THF-soluble lignin fractions, and large THF-insoluble lignin fragments and the input variables in coded units.

#### 3.2.1 Effect of Variables on Monomer Yield

The yield of the monomers preferred a continuous time profile according to the information criteria in Table S2. However, the estimate of the variance components of the random intercept *a*_i_ was 0, while the categorical time profile in eq 5 had a non-zero variance component. The result that a more restricted fixed effect model (eq 4) did not show heterogeneity in contrast to the more elaborative fixed effect model (eq 5) may indicate that the preferred model is oversimplified. The cumbersome analysis of the GC–MS spectra with the related product peak identification for 108 analytical experimental data may hamper the relevant data extraction especially for very high temperatures. This might have affected the data quality with the inclusion of some technical noise and the misidentification of certain compounds, which might have reduced the observed heterogeneity and consequently interfered with the estimation of the random intercept. Furthermore, as the difference in the information criteria between the two time profiles is relatively small, we preferred to optimize the more general time structure to address the heterogeneity.

The LMM-RSM analysis in Table S2 shows that the monomer yield is positively affected by the variable temperature with a quadratic dependence (*T*^2^), especially at later stages of the reaction (i.e., temperature–time interaction; (*T* × *t*)). Similarly, the particle size also has a moderate effect on the yield with larger particle sizes having a clear negative effect on the yield. Lignin loading tends to reduce the amount of the expected monomers produced during the reaction, especially at later stages of the reaction (*T* × *L* × *t*). This is mainly associated with a reduced solvolytic efficacy of ethanol at high lignin loadings and the repolymerization of lignin fragments, which is associated with the prolonged reaction and high temperatures in the absence of a reducing agent such as hydrogen. At high lignin loadings, the amount of insoluble fragments increases, leading to catalyst fouling and deactivation, leading also to insufficient hydrogen production from ethanol reforming (Figure 4a). Temperature and catalyst concentration have a significant negative interaction, meaning that the larger the catalyst concentration, the lower is the effect of temperature on the yield of monomers.

**Figure 7:**
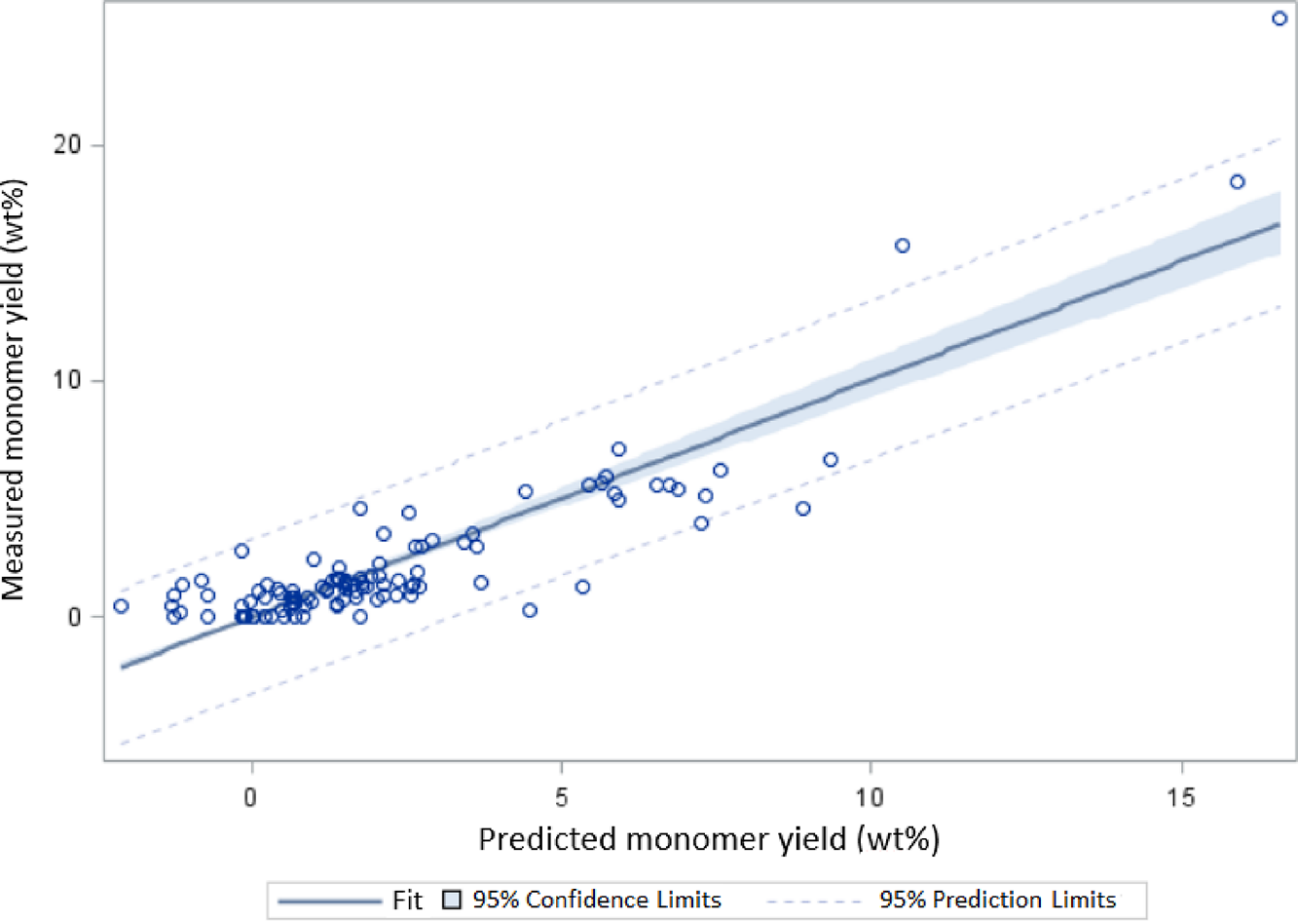
Observed vs predicted monomer yield.

The relationship between the predicted and the observed monomer yield is strong, as shown in Figure 7. The variability explained by the fixed effects of the model is relatively high, *i.e.*, *R*^2^ = 0.78, which ensures a satisfactory adjustment of the polynomial model to the experimental data. Moreover, the Shapiro–Wilk test could not reject the normality hypothesis of the residuals (*p* = 0.84), suggesting that the underlying assumptions of the normality of the residuals are appropriate.

#### 3.2.2 Effect of Variables on the Yield of THF-Soluble Fragments

**Figure 8:**
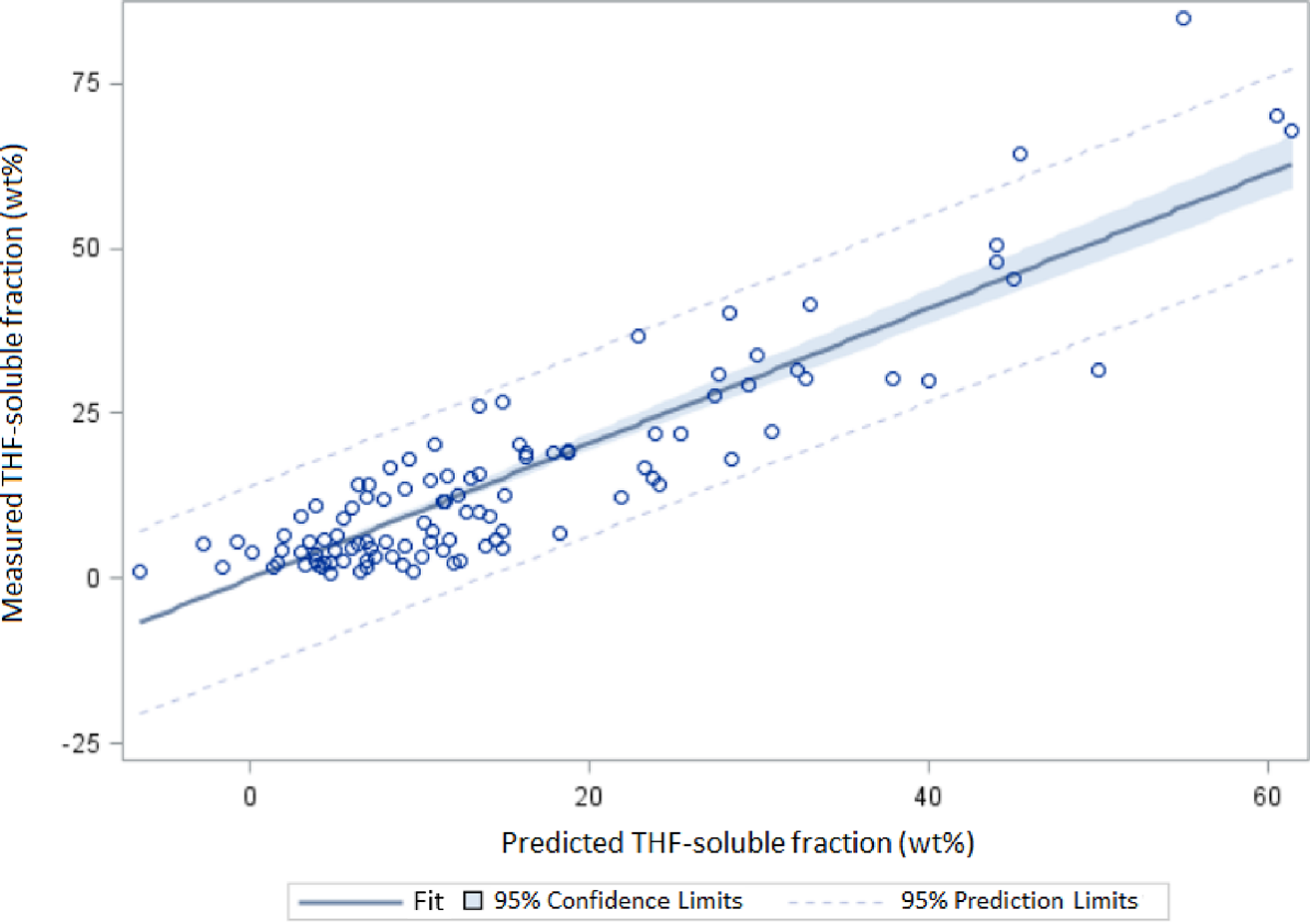
Observed vs predicted yield of THF-soluble fragments.

For this process output stream, the goodness of fit measures indicated a preference for a continuous time profile. The results of the LMM-RSM analysis summarized in Table S2 indicate that the yield of THF-soluble fragments exhibits a positive dependence with temperature with a quadratic dependence, to a much larger degree compared to the lignin monomers. Lignin loading is definitely a significant process parameter for the evaluation of the yield of the soluble fragments since it appears with significant effect sizes in the model as linear and quadratic effects as well as having an interaction with temperature, particle size (with negative effect sizes), time and temperature, and time and particle size. Remarkably, the linear effect size is much larger than the linear negative effect size compared to the one estimated for monomers. Larger catalyst particle sizes are responsible for an increase in the yield for soluble fragments. Time interferes with a quadratic relationship, whereas catalyst concentration contributes negatively to the yield of the soluble fragments. The day-to-day variability, which is estimated through the random intercept, has in this case a remarkable size. Also, for this response variable, there is good agreement between predicted and observed responses, as depicted in Figure 8 (*R*^2^ = 0.81). The Shapiro–Wilk test does not reject the null hypothesis of the normality of the residuals (*p* = 0.97).

#### 3.2.3 Effect of Variables on the Yield of THF-Insoluble and Char Fragments

**Figure 9:**
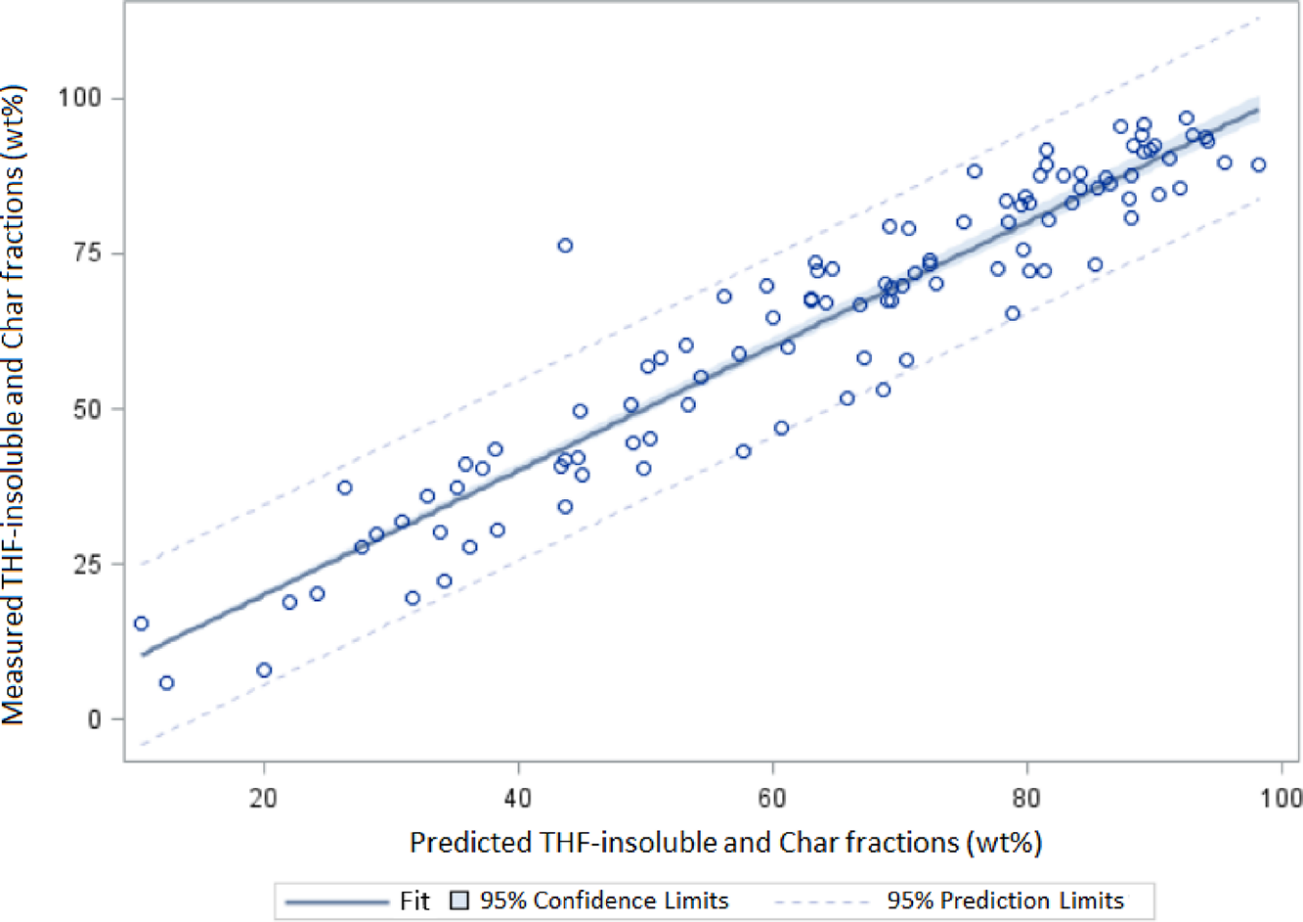
Observed vs predicted yield of THF-insoluble and char fractions.

The goodness of fit measures preferred a categorical time profile. Surprisingly, particle size does not interfere with char formation. Temperature and lignin loading contribute to char formation with a quadratic dependence, especially for high temperatures. Catalyst concentration is also a significant agent in interactions with temperature. This product stream also shows an interesting interaction effect between reaction time and temperature. But, this effect is decreasing over time: higher temperatures at early stages of the reaction may be responsible for larger amounts of char than at later stages of the reaction, especially when high lignin loadings are used. The variance component addressing the heterogeneity in the randomization process is of a moderate but non-negligible size. Figure 9 supports the very high significance of the model, with very good agreement between predicted and observed responses (*R*^2^ = 0.95). Also, the normality of the residuals could not be objected by the Shapiro–Wilk test (*p* = 0.98).

## 4 Conclusions

**Figure 10:**
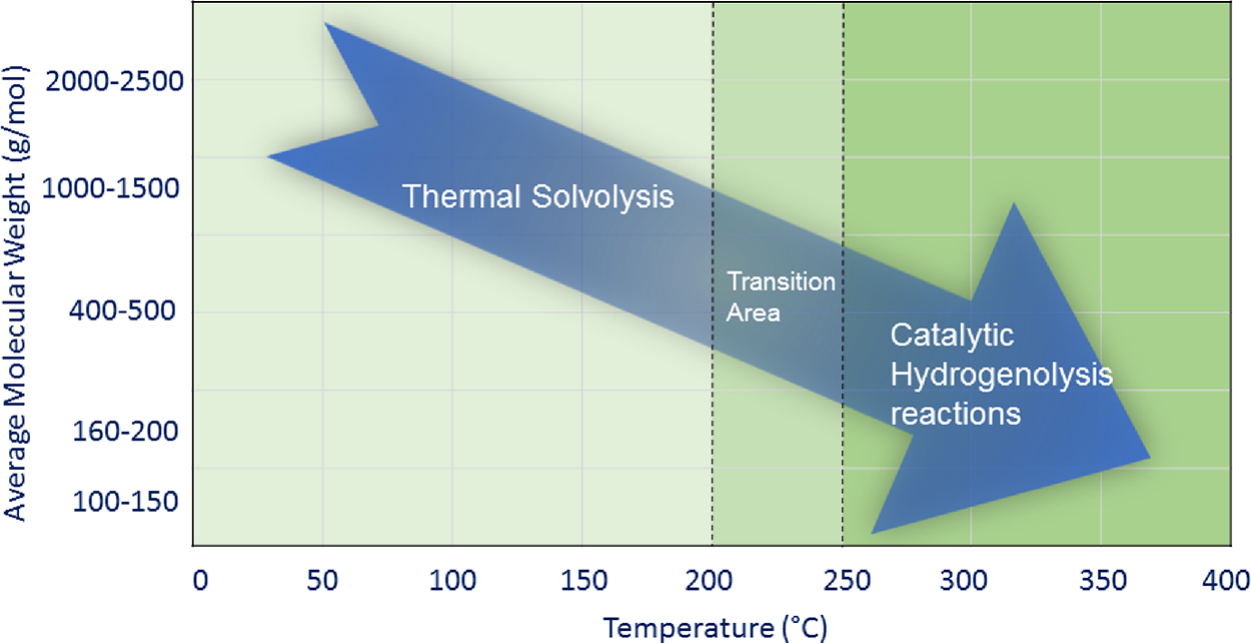
Process stages during ethanol-mediated catalytic depolymerization of technical lignin.

In this work, the performance of catalytic depolymerization of lignin was evaluated by monitoring three output variables over time: (i) monomer yield, (ii) yield of THF-soluble fragments, and (iii) yield of THF-insoluble fragments and char. For this purpose, we considered four input process variables (reaction temperature, lignin loading, catalyst concentration, and catalyst particle size). We presented a structured experimental design for this purpose. The large number of experiments were combined in experimental runs to reduce the processing time. In particular, the resulting day-clustered randomization can be considered as a particular case of incomplete block design with some additional constraints imposed by time. Despite the fact that we miss a solid mathematical representation for this specific design, the use of an LMM instead of a standard ANOVA model (with the additional random term to address the day-to-day heterogeneity) can be seen as a first attempt toward a more advanced analysis, which also finds support in the literature.^16^ Our qualitative report of the experimental results is in line with previous work by Kouris *et al*.^12^ and is further supported by the LMM-RSM quantitative analysis. The three models showed a high prediction quality, and all identified a non-negligible day-to-day variability due to the specific randomization of the experiment. More importantly, a significant negative solubility effect was found at increased lignin loadings, resulting in a reduced depolymerization degree due to a decreased solubility of lignin in the solvent. Additionally, higher lignin loadings emphasized the dominating condensation reactions at 250 °C, yielding significant amounts of char. The adsorbed species on the catalyst surface at low reaction temperatures (120–250 °C) were generally depolymerized products, indicating that desorption of products from the catalyst surface required high temperatures in an environment of high lignin concentrations. An increased catalyst concentration enlarged this phenomenon. The yields of depolymerized products (monomers and oligomers) were highest at a reaction temperature of 340 °C, confirming the high rates of depolymerization and repolymerization suppression reactions (i.e., alkylation, Guerbet, and esterification) at this temperature. At a reaction temperature of 120 °C, the dissolved products in the reaction mixture were mainly of high molecular weight (∼6000–7000 g/mol), indicating condensation of reactive lignin fragments. Overall, the one-step catalytic depolymerization of lignin in the presence of ethanol comprises two main steps, as shown in Figure 10. At lower temperatures, in the range of 120–200 °C, lignin is solubilized (solvolysis step) and some bonds are cleaved, leading to lignin fragments with a lower molecular weight (*M*_W_) in the range of 400–900 g/mol. Ethanol is a solvent with high polarity and hydrogen bonding ability for lignin solvolysis. Thermolytic cleavage of weak β-O-4 ether bonds, which according to the literature^26^ can already occur at a relatively mild temperature of 200 °C, can result in thermal solvolysis of lignin and explain the molecular weight reduction. Cracking of these fragments, via hydrogenolysis reactions, using a catalyst requires temperatures higher than 250–300 °C (catalytic hydrogenolysis step), where hydrogen formation through ethanol reforming is enhanced. An inherent problem during these steps, especially when high lignin loadings are applied, is the absorption of lignin fragments on the heterogeneous catalyst surface, leading to severe deactivation.

## Supporting Information Available

Final LMM-RSM model equations for the three outputs after backward elimination based on the coded input factors and gas phase analysis for assessing the hydrogen production at various reaction temperatures and reaction times (PDF)
