# Supplementary material for: Optimizing Catalytic Depolymerization of Lignin in Ethanol with a Day-Clustered Box–Behnken Design
Source: Ind Eng Chem Res. 2023 Apr 28;62(18):6874–85. doi: 10.1021/acs.iecr.2c03618 (PMC10241191; doi:10.1021/acs.iecr.2c03618)
Supplement: Supplementary file 1 — ie2c03618_si_001.pdf [file ie2c03618_si_001.pdf]

Supporting Information

Optimizing Catalytic Depolymerization of Lignin in Ethanol with a day-clustered Box-Behnken Design

Panos D. Kouris[1(†)], Alberto Brini[2(†)], Eline Schepers[1], Michael D. Boot[1], Edwin R. van den Heuvel[2], Emiel J.M. Hensen[1,*]

[1] Laboratory of Inorganic Materials and Catalysis, Department of Chemical Engineering and Chemistry, Eindhoven University of Technology, Eindhoven, The Netherlands

[2] Department of Mathematics and Computer Science, Eindhoven University of Technology, Eindhoven, Netherlands

[(†)] Joint first authors

[*] Correspondence to: e.j.m.hensen@tue.nl

Contents of the supporting Information:

Total number of tables: 2

Supplementary Tables

Table S1. Analysis of the gas phase after catalytic lignin ethanolysis at 120 °C, 250 °C and 340 °C for various reaction times (0, 2, 4 and 8 hours), lignin:ethanol ratio of 1:7 (g/mL), catalyst particle size of 125 µm and catalyst concentration (g/g lignin) of 0.4375.

**Gases**	**Concentration (g/mL)**			
	**Reaction temperature 120 °C**			
	**0 h**	**2h**	**4h**	**8h**
Hydrogen	15	0	54	0
C1-C4 hydrocarbon gases	13	1	1	2
	**Reaction temperature 250 °C**			
	**0 h**	**2h**	**4h**	**8h**
Hydrogen	162	198	182	165
C1-C4 hydrocarbon gases	33	98	157	208
	**Reaction temperature 340 °C**			
	**0 h**	**2h**	**4h**	**8h**
Hydrogen	194	465	369	319
C1-C4 hydrocarbon gases	1978	2586	3969	4901

Table S2. Final LMM-RSM model for the 3 outputs after backward elimination based on the coded input factors, where t is the reaction time, T is reaction temperature, L is lignin loading, P is particle size and C is catalyst concentration.

Output variable	Final Model
**Monomer yield**	$0.89 + 2.72T - 0.16L + 0.09C + 0.18P + 0.06(T \times L) + 0.08(T \times C) + 2.00T^2 + 0.44P^2$ $(-0.50t - 1.27(T \times t) - 0.001(L \times t) - 0.26(C \times t) + 0.38(P \times t) + 0.82(T^2 \times t) - 0.02(P^2 \times t) - 0.22(T \times L \times t) - 0.13(T \times C \times t))\mathbb{I}_{t=0} +$ $(0.64t + 2.42(T \times t) - 0.63(L \times t) - 0.84(C \times t) + 0.23(P \times t) + 2.82(T^2 \times t) - 2.65(P^2 \times t) - 3.46(T \times L \times t) - 4.22(T \times C \times t))\mathbb{I}_{t=2} +$ $(-0.08t + 0.84(T \times t) - 0.49(L \times t) - 0.06(C \times t) + 0.08(P \times t) + 1.00(T^2 \times t) - 0.84(P^2 \times t) - 2.31(T \times L \times t) + 0.16(T \times C \times t))\mathbb{I}_{t=4} +$ $a_i + e_i,$ $a_i \sim N(0,0.14)$ $e_i \sim N(0,4.14)$
**Yield of THF-soluble fragments**	$5.59 - 1.56t + 10.45T - 4.60L - 6.70C + 0.84P + 0.84(T \times t) + 1.01(L \times t) + 0.24(C \times t) + 0.14(P \times t) - 7.24(T \times L) + 8.99(T \times C) - 6.06(C \times P) - 9.03(L \times P) + 16.56T^2 + 5.50L^2 + 0.17t^2 + 1.80(T \times L \times t) - 6.45(T \times C \times t) + 5.38(L \times P \times t) + 4.77(C \times P \times t) + 0.63(T \times C \times t^2) - 0.47(L \times P \times t^2) - 0.52(C \times P \times t^2) + a_i + e_i,$ $a_i \sim N(0,53.38)$ $e_i \sim N(0,69.63)$
**Yield of THF-insoluble fragments and Char**	$82.38 - 19.51T + 12.73L + 11.77C - 8.37(T \times C) - 33.660T^2 - 5.39L^2$ $(0.08t + 16.00(T \times t))\mathbb{I}_{t=0} +$ $(4.89t + 4.74(T \times t))\mathbb{I}_{t=2} +$

	$\left(1.75t + 0.28(T \times t)\right)\mathbb{I}_{t=4} +$
	$a_i + e_i,$
	$a_i \sim N(0,17.51)$
	$e_i \sim N(0,67.44)$
